# Prospective clinical trials on correlations between macro-/ microelements and vitamins and cancer risk

**DOI:** 10.1186/1897-4287-10-S3-A15

**Published:** 2012-04-20

**Authors:** Jan Lubinski

**Affiliations:** 1 Read-Gene SA and Pomeranian Medical University, Szczecin, Poland

## 

There is no doubt that several macro-/microelements and vitamins are key features to prevent malignancies in experimental studies on animals. Literature data and results of our own studies are strongly suggesting that for malignancies characterized by strong contribution of inflammatory response to environmental carcinogens such as lung, colorectal or prostate cancers, penetrance level can be reduced significantly by selenium supplementation in sub-group of persons with Se level < 100 µg/l of serum/plasma. Thus, almost entire Polish population should be supplemented with Se. It seems that the very important exception are females with increased risk of breast/ovarian cancers, because only 40% carries selenoprotein genotypes in which supplementation can be recommended as the option. Our center has been realizing prospective studies validating the above findings and searching for identification of similar associations for the large panel of macro-/microlements and vitamins. The goal is to collect biological samples (at least DNAs and sera) from more than 20,000 persons, mainly with diagnosed increased risk of cancers. The whole group will be followed for at least 5 years. Bio-banking and collection of medical information will be performed annually.

Basic selenium level will be measured for majority of participants. Selected participants matching requirements of other projects will get also results of analyses of other macro-/microelements and vitamins.

